# The susceptibility of sea-island cotton recombinant inbred lines to *Fusarium oxysporum* f. sp. *vasinfectum* infection is characterized by altered expression of long noncoding RNAs

**DOI:** 10.1038/s41598-019-39051-2

**Published:** 2019-02-27

**Authors:** Zhengpei Yao, Quanjia Chen, Dong Chen, Leilei Zhan, Kai Zeng, Aixing Gu, Jian Zhou, Yu Zhang, Yafu Zhu, Wenwei Gao, Liping Wang, Yi Zhang, Yanying Qu

**Affiliations:** 1 College of Agronomy, Xinjiang Agricultural University, Urumqi, 830052 China; 2 Center for Genome Analysis, ABLife Inc., Optics Valley International Biomedical Park, East Lake High-Tech Development Zone, 388 Gaoxin 2nd Road, Wuhan, Hubei 430075 China; 3 Crop Research Institute, Xinjiang Academy of Agricultural Reclamation, Shihezi, 832000 China

## Abstract

Disease resistance is one of the most complicated yet important plant traits. The potential functions of long noncoding RNAs (lncRNAs) in response to pathogenic fungi remain unclear. In this study, we sequenced the transcriptomes of four different sea-island cotton (*Gossypium barbadense*) recombinant inbred lines (RILs) with susceptible, highly susceptible, highly resistant, or super highly resistant phenotypes and compared their responses to *Fusarium oxysporum* f. sp. *vasinfectum* (*Fov*) infection with those of their susceptible and resistant parents. Infection-induced protein coding genes were highly enriched in similar disease resistance-related pathways regardless of fungal susceptibility. In contrast, we found that the expression of a large number of *Fov* infection-induced lncRNAs was positively correlated with plant susceptibility. Bioinformatics analysis of potential target mRNAs of lncRNAs with both trans-acting and cis-acting mechanisms showed that mRNAs co-expressed or co-located with *Fov*-regulated lncRNAs were highly enriched in disease resistance-related pathways, including glutathione metabolism, glycolysis, plant hormone signal transduction, anthocyanin biosynthesis, and butanoate metabolism. Together these results suggest that lncRNAs could play a significant role in the response to pathogenic fungal infection and the establishment of disease resistance. The transcriptional regulation of these infection-susceptible lncRNAs could be coordinated with infection-susceptible mRNAs and integrated into a regulatory network to modulate plant-pathogen interactions and disease resistance. *Fov*-susceptible lncRNAs represent a novel class of molecular markers for breeding of *Fov*-resistant cotton cultivars.

## Introduction

Long noncoding RNAs (lncRNAs), the most common noncoding RNAs, are transcripts that are longer than 200 nt. LncRNAs have a low conservation rate, low expression levels, tissue- and cell-specific expression patterns, and highly complex and diverse gene regulatory mechanisms and are associated with every biological process (BP) in virtually all eukaryotic organisms^1^. In general, lncRNAs influence the physiological and biochemical processes of organisms by acting as molecular signals, decoys, guides, or scaffolds^2^. The biological importance of lncRNAs has stimulated significant research interest in recent years. Transcriptome sequencing and computational methods have allowed for the systematic identification and classification of lncRNAs in many different species, including mammals and plants^3^.

Disease resistance represents one of the most important biological traits for breeding crop cultivars around the world^4^ and has always been a vital component of cultivation method development^5^. However, diseases caused by fungi remain a major threat to crops^6–9^. Rapid generation of superoxide and accumulation of H_2_O_2_, called an oxidative burst, is an early feature of the hypersensitive response when plants sense pathogens. Oxidants act as direct protective agents, substrates for oxidative cross-linking in the cell wall, triggers of hypersensitive cell death, and diffusible signals to trigger protective gene expression in surrounding cells^10–13^. The signal network coordinated by reactive oxygen intermediates contributes to the establishment of plant immunity^14–17^. Recent studies have revealed that lncRNAs are important components of anti-fungal networks in plant immunity^18,19^. LncRNAs are involved in resistance to *Verticillium dahliae*, a fungal pathogen of cotton^20^, which causes verticillium wilt (VW). Moreover, it has been suggested that lncRNAs of *Arabidopsis thaliana* and bananas (*Musa acuminata*) respond to *F*. *oxysporum* infection^21,22^ and could play important roles in anti-fungal networks.

*Fusarium oxysporum* f. sp. *vasinfectum* (*Fov*) is a soil-borne plant fungal pathogen that causes vascular wilt disease through root infection in a wide range of plants, including economically important crops such as cabbage, banana, cotton, flax, watermelon, chickpea, and tomato^23–29^. These pathogens can exist in the form of mycelia, chlamydospores, or microsclerotia in soil and crop debris and can persist in the soil for 5–10 years before causing recurrent infection^27^. Fusarium wilt (FW) frequently causes disease symptoms after the seedling stage that peak at the squaring stage. Its visible symptoms include leaf yellowing, wilting, vascular tissue damage, and ultimately plant death^24^. Management of FW is achieved mainly by the use of chemical fungicides; however, these affect soil health and their efficiency is often limited by pathogen variability^30^. Therefore, the breeding of disease-resistant cultivars remains the primary control method for crops, and the breeding of FW-resistant cotton cultivars is of great importance^31^.

In the past 50 years, substantial progress has been made in cotton breeding to progressively increase resistance to FW. A few varieties of Upland cotton (*Gossypium hirsutum*) with resistance to *Fov* are commercially available, but no *Fov*-resistant sea-island cotton (*Gossypium barbadense*) varieties. Sea-island cotton has high values owing to its unprecedented quality, fiber length, fitness, and strength and accounts for 5–8% of worldwide cotton production.

To better characterize the molecular markers and genes related to *Fov* resistance in sea-island cotton, we performed hybrid experiments using cultivar 06-146 with high resistance to FW as the male parent and cultivar Xinhai-14 with high susceptibility to FW as the female parent. We then obtained recombinant inbred line (RIL) populations and assessed their *Fov*-susceptibility in the field over successive generations. We continued to select offspring that were more susceptible or more resistant than their parents. We chose two resistant offspring and two susceptible offspring among F_6_ RILs, planted the seeds in a greenhouse, and sequenced the root transcriptomes of the parents and the RIL offspring 40 h after *Fov* infection. The availability of the complete genome sequences of *G*. *barbadense*^32^ and *G*. *hirsutum*^33,34^ allowed us to fully analyze the protein-coding genes that respond to *Fov* infection. We found that expression of genes highly enriched in pathways related to the oxidative burst were induced regardless of *Fov* susceptibility. We further identified lncRNAs from these transcriptomes and found that induction of lncRNA expression characterized the susceptibility of the RIL offspring to *Fov* infection. These findings underline the importance of lncRNAs in fungal infection and suggest that lncRNAs could be important markers for breeding *Fov*-resistant cultivars.

## Results

### Symptoms of infected cotton

Previous studies have shown that *G*. *barbadense* is more resistant to VW than *G*. *hirsutum*^20,35^ but less resistant to *Fov*^36^. However, few studies have examined how *G*. *barbadense* responds to FW. Therefore, we obtained and crossed two *G*. *barbadense* cultivars with different levels of resistance to *Fov* infection, including a resistant cultivar (paternal plant, 06-146, [F]) and a susceptible cultivar (maternal plant, Xinhan-14, MX-14, [M])^37^. In the F_6_ RILs, four lines emerged based on their level of resistance, including two resistant (super highly resistant [SHR] and highly resistant [HR]) and two susceptible (highly susceptible [HS] and susceptible [S]) lines. In general, disease symptoms appear 4–7 days after inoculation. However, plant immune responses and transcriptional changes occur early during *Fov* infection^35,38^.

In order to profile plant transcriptional responses to *Fov* infection, seedlings at the one true-leaf stage were inoculated with *Fov*. Lower hypocotyls of cotton seedlings were then collected to assess responses to infection. A quantitative real-time PCR (qRT-PCR) assay was used to assess the expression of the *Fov* infection-related gene *FOTG*, and we found evidence of infection 28 h after inoculation (Supplementary Fig. S1). We then extracted total RNA at a later time point, 40 h after inoculation, for RNA-seq experiments to assess the transcriptional response to *Fov* infection. We did not observe any symptoms 40 h after inoculation (Supplementary Fig. S2). Previous studies have examined the growth of *Fov* in the lower hypocotyls of different plants within the first two days post infection (dpi). During this period of infection, green fluorescent protein-expressing strains of *Fov* were shown to grow on the root epidermis and adhere to the junctions of epidermal cells in tomato root^39^ and melon^40^. In a study of *Fov*-infected cotton, it was shown that pathogens penetrate the roots 1 dpi, enter the vascular system, and move up through the plant to the hypocotyl vascular tissue 2–3 dpi, where infection causes vascular browning^38^. We observed vascular browning at a similar time point as this previous report. Based on these results, we anticipated that *Fov* infection would cause a transcriptional response in the lower hypocotyls 40 h after inoculation.

### Altered expression profiles of protein-coding genes after *Fov* infection

**Figure 1:**
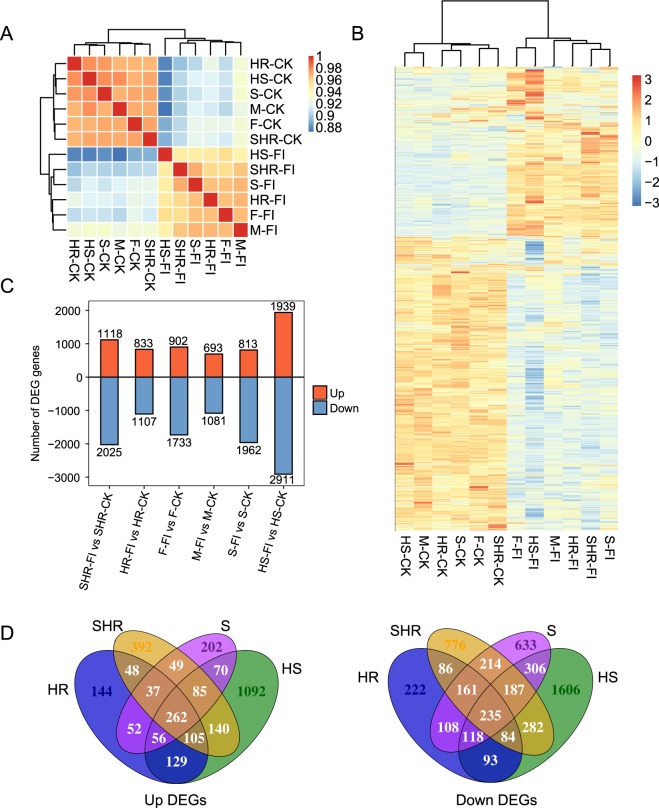
Assessment of the global characteristics of the response of sea-island cotton mRNA expression to *Fov* infection by RNA-seq. (**A**) Hierarchical clustering heatmap showing the distinct expression patterns of infected and control samples. (**B**) Hierarchical clustering heatmap showing the expression patterns of DEGs. (**C**) Barplot showing the distribution of DEGs in each group. (**D**) Venn diagram showing co-upregulated (left panel) and co-downregulated (right panel) DEGs in the four RILs.

In the current study, we explored the transcriptional profiles of protein-coding genes that respond to *Fov* in different genotypes of the same species. Twelve RNA libraries of *Fov* infected (FI) and control check (CK) plants, including two parents [M-FI and M-CK; F-FI and F-CK] and four RILs [HR-FI and HR-CK; SHR-FI and SHR-CK; S-FI and S-CK; and HS-FI and HS-CK], were constructed by pooling RNA isolated from three different individuals. Through Illumina HiSeq2000 sequencing, we generated over 350 million pair-end reads corresponding to an average of 29.4 million sequencing reads per sample (Supplementary Table S1). Using TopHat2 alignment software^41^, 81.6% of all reads were successfully mapped to the current Upland cotton reference genome^34^. Global clustering analysis of Pearson’s correlation coefficients between each sample pair revealed separate transcriptome patterns between control and infected groups (Fig. 1A). Differentially expressed gene (DEG) analysis (*p* value < 0.01 and |log_2_ fold change (FC)| > 2) was performed to identify *Fov*-regulated genes in each pair of the infected and control RNA-seq samples for the two parental and four RIL lines. Clustering heatmaps of DEGs demonstrated a clear separation between control and infected groups, with most genes being downregulated (Fig. 1B). Upregulated and downregulated genes were consistent among infected and control genotypes, suggesting that the transcriptional response of mRNAs to *Fov* infection was highly conserved among the six genotypes (Fig. 1B). The number of DEGs was highest in the HS-FI versus HS-CK comparison (Fig. 1C). We detected 337 co-upregulated and 491 co-downregulated DEGs between the M-FI versus M-CK and F-FI versus F-CK comparisons, respectively, representing 26.79% and 22.15% of the upregulated and down-regulated DEGs, respectively (Supplementary Fig. S3A). Analysis of the four RILs revealed similar results (Fig. 1D). These results suggested all the parental and RIL lines respond to *Fov* infection similarly, although substantial differences were presented as well.

EdgeR and other statistical pipelines were initially designed to assess RNA-seq data with replicates, although edgeR can also be used to assess RNA-seq data without replicates. Therefore, we used Chi-square test to analyze DEGs and compared the results with those of edgeR analysis. As shown in Supplementary Table S2, we found that the Chi-square test detected more differentially expressed (DE) mRNAs than edgeR in all groups except the HS group. The percentage of overlapping DEGs between edgeR and Chi-square tests was less than 30% of the total number of DEGs identified by the two methods, suggesting that differences exist between them.

**Figure 2:**
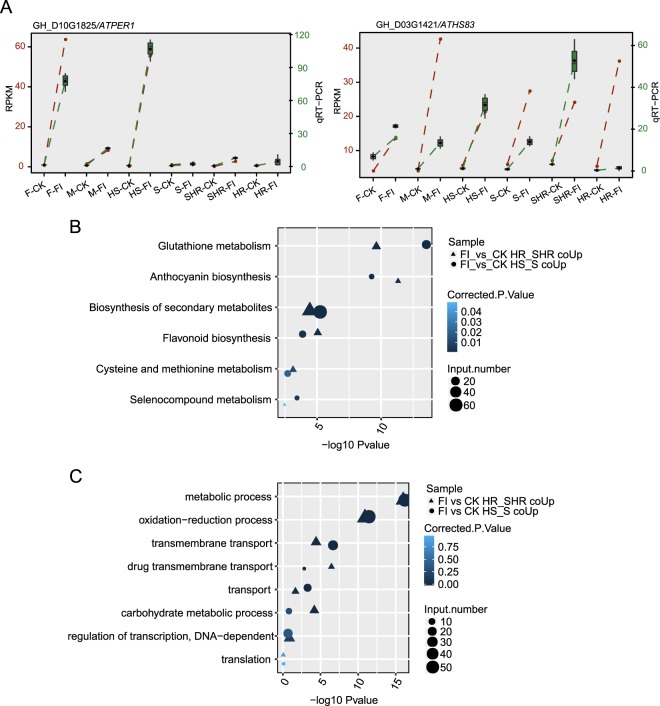
qRT-PCR validation and functional enrichment analysis of co-DEGs in the four RILs. (**A**) Line plot showing consistent changes in expression between RNA-seq (FPKM) and qRT-PCR for two selected DEGs. *ACT7* was used as an internal control to obtain the relative expression level of each DEG in the qRT-PCR experiment. Three replicates were used for qRT-PCR experiments. (**B**) Enriched functional KEGG pathways of co-upregulated DEGs in resistant and susceptible groups. Colors represent corrected *p* values; the size of the circle or triangle represents the number of genes in each term or pathway; the shape represents the two co-DEG groups, which were the same in (**C**). (**C**) Enriched functional BP terms of the co-upregulated DEGs in resistant and susceptible groups.

We performed qRT-PCR experiments to validate the expression levels of DEGs. We selected six DEGs with elevated expression levels and one with reduced expression level in the HS line after infection. The *Fov*-regulated expression of all seven genes were well-detected by qRT-PCR analysis in the HS line (Fig. 2A, Supplementary Fig. S3B). We also found that *Fov*-upregulated expression of G1421, G1208, and G2549 were evident in almost all other groups. However, *Fov*-regulated expression of the other four protein-coding genes were either small or inversed in many other groups (Fig. 2A, Supplementary Fig. S3B).

To further explore the functions of DEGs, we performed functional clustering of upregulated genes and found similar enriched pathways among all four RILs (Supplementary Fig. S4). Anthocyanin biosynthesis, phenylpropanoid biosynthesis, biosynthesis of secondary metabolites, and phenylalanine metabolism were highly enriched among the upregulated DEGs (Supplementary Fig. S4). Similar Kyoto Encyclopedia of Genes and Genomes (KEGG) pathways were also enriched among downregulated DEGs, suggesting that expression of these genes was deregulated by *Fov* infection. DNA replication and plant hormone signal transduction were also enriched among downregulated DEGs (Supplementary Fig. S4). We also obtained 452 co-upregulated DEGs in the two resistant groups and 473 co-upregulated DEGs in the two susceptible groups (Fig. 1D). Enriched KEGG pathways included glutathione metabolism, anthocyanin biosynthesis, flavonoid biosynthesis, biosynthesis of secondary metabolites, cysteine and methionine metabolism, and selenocompound metabolism (Fig. 2B). Many of these pathways have been associated with the response to fungal infection in plants. For example, flavonoid glycoside has been reported to exhibit antifungal activity against different *Fusarium oxysporum* f. sp. *dianthi* pathotypes^42^. Enriched BP terms in the Gene Ontology (GO) database also involved similar functions (Fig. 2C). Metabolic process, oxidation-reduction process, and transmembrane transport-related terms were most highly enriched (Fig. 2C), consistent with the important role of the oxidative burst in pathogen resistance.

### Genome-wide identification and characterization of lncRNAs

A previous study revealed that lncRNAs play important roles in responding to VW in cotton^20^. Using an integrated approach, we identified 11,336 predicted lncRNAs from the 12 transcriptome datasets (Supplementary Table S3). Of these putative lncRNAs, 81.0% were long intergenic noncoding RNAs (lincRNAs), 14.4% were intronic lncRNAs, and 4.6% were natural antisense lncRNAs. In total, 63.9% and 36.1% of lncRNAs were distributed on the At and Dt subgenomes, respectively.

**Figure 3:**
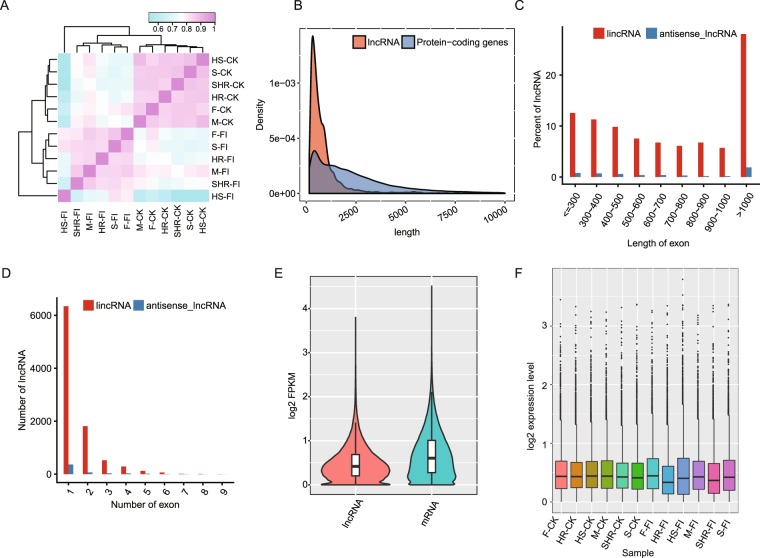
Characterization of predicted lncRNAs in all *G*. *barbadense* genotypes. (**A**) Hierarchical clustering heatmap showing the distinct lncRNA expression patterns of infected and control samples. (**B**) Length distribution of all lncRNA and mRNA transcripts. (**C**) Barplot showing the length distributions of lincRNAs and antisense lncRNAs. (**D**) Distribution of lincRNA exons and antisense lncRNAs. (**E**) Violin plot showing lower expression levels of lncRNAs compared with mRNAs. (**F**) Boxplot showing the FPKM distribution of cotton lncRNAs before and after *Fov* infection.

The global expression pattern of lncRNAs also differed between control and infected groups (Fig. 3A), demonstrating that *Fov* infection also greatly alters the expression pattern of lncRNAs. The mean length of lncRNA transcripts was shorter than that of protein-coding transcripts (1042.1 nt for lncRNAs and 3355.48 nt for protein-coding transcripts; Fig. 3B). The lengths of lncRNAs ranged from 201–89,370 nt, but more than 76.4% of lncRNAs were between 200 and 1000 nt in length (Fig. 3C). Consistent with previous studies^20,21^, approximately 69.2% of lncRNAs had only one exon, and 30.8% had multiple exons (Fig. 3D). Calculated lncRNA expression levels were found to be lower than mRNA expression levels (*t*-test *p* value = 0, Fig. 3E). From these results, the predicted lncRNA profile was highly consistent with other studies, supporting the reliability of our prediction pipeline. From the boxplots of the 12 samples, no difference was found in the CK group, whereas a non-consistent expression pattern was found for the FI group (Fig. 3F). The two resistant samples showed downregulated expression after *Fov* infection (Fig. 3F). Inspection of global expression levels normalized to fragments per kilobase per million mapped reads (FPKM) for all mRNAs and lncRNAs indicated that the expression levels of most lncRNAs were lower than 10 FPKM (Fig. 3E,F).

### Exploration of pathogen-induced DE lncRNAs in parental cultivars

**Figure 4:**
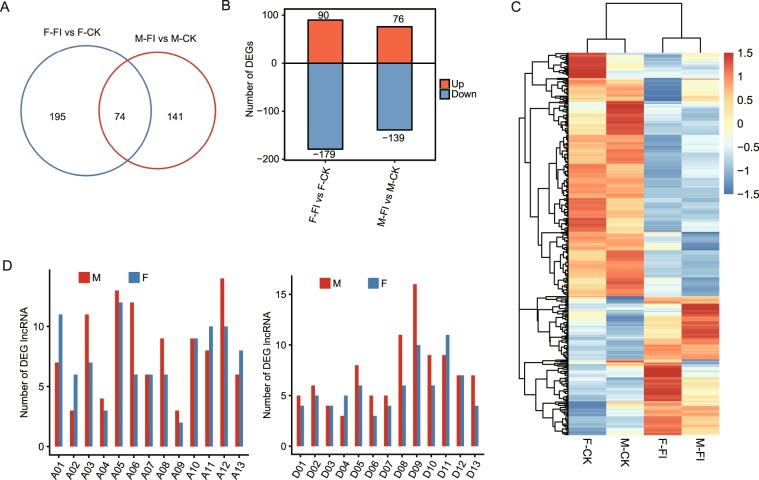
DE lncRNAs in two *Fov*-infected parental cultivars. (**A**) Venn diagrams of DE lncRNAs showing M-FI versus M-CK and F-FI versus F-CK comparisons. (**B**) The number of upregulated and downregulated DE lncRNAs. (**C**) Heatmaps of DE lncRNAs from M-FI versus M-CK and F-FI versus F-CK comparisons. The heatmap was generated from hierarchical analysis of DE lncRNAs. (**D**) Comparisons of the distribution of DE lncRNAs on chromosomes from the At and Dt subgenomes in two parental cultivars.

Because the two parental cultivars came from different genetic backgrounds and had different resistance levels, we hypothesized that they would demonstrate different responses to *Fov* infection. Using the edgeR^43^ package of R software, 215 and 268 DE lncRNAs (false discovery rate [FDR] ≤ 0.05; |log2FC| ≥ 1) were found in the M-FI versus M-CK and F-FI versus F-CK comparisons, respectively. Although the number of DE lncRNAs was similar between these two groups, only 74 common DE lncRNAs were identified (Fig. 4A), indicating that lncRNA expression in response to *Fov* infection in these two cultivars was different. In both comparisons, *Fov* infection decreased expression of lncRNAs (Fig. 4B,C). By comparing DEGs and DE lncRNAs, we also detected more downregulated mRNAs in the M-FI versus M-CK and F-FI versus F-CK comparisons (Fig. 1C). As more DEGs and DE lncRNAs were detected in the paternal group, we concluded that this group was more sensitive to *Fov* infection.

The distribution of DE lncRNAs on chromosomes was also investigated. In M-FI versus M-CK and F-FI versus F-CK comparisons, DE lncRNAs were distributed on every chromosome. However, the lncRNA density (i.e., count by chromosome length) was not uniform across the two subgenomes. More DE lncRNAs were found on chromosomes A03, A05, A06, A12, D08, and D09 for the M-FI versus M-CK comparison (Fig. 4D), whereas more lncRNAs were found on chromosomes A01, A05, A11, A12, D09, and D11 for the F-FI versus F-CK comparison (Fig. 4D). This suggests that location bias plays a role in lncRNA induction by *Fov* infection.

### Identification of pathogen-induced DE lncRNAs in RILs

**Figure 5:**
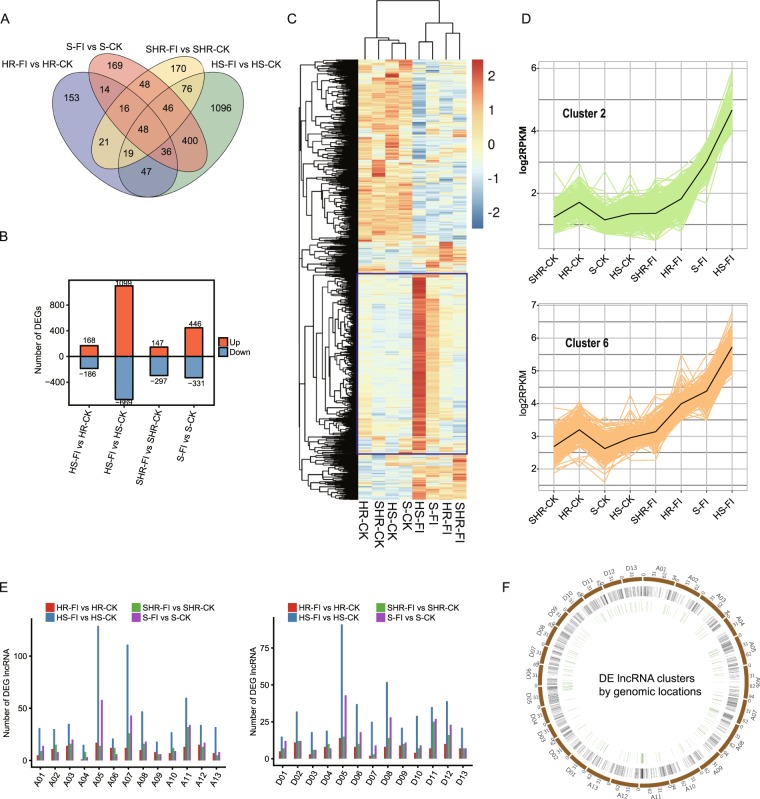
Expression of DE lncRNAs in four *Fov*-infected F_2:6_ RILs. (**A**) Venn diagram showing the few overlapping DE lncRNAs from the four comparisons. (**B**) The number of upregulated and downregulated DE lncRNAs from the four comparisons. (**C**) Hierarchical clustering heatmap of all DE lncRNAs from the four comparisons. lncRNAs in blue boxes gradually decreased with resistance level. (**D**) k-means clustering analysis revealed two clusters with consistently increasing expression level in RILs with decreasing resistance levels. The black line indicates the mean expression value of genes in each cluster. (**E**) Barplot showing the distribution of DE lncRNAs on chromosomes from At and Dt subgenomes in four RILs. (**F**) Circos plot showing the genomic distribution of lncRNA clusters by distance. The green circle represents lncRNA clusters; the black circle represents DE lncRNAs; the brown circle represents the chromosome.

We analyzed pathogen-induced DE lncRNAs in the four RILs with a similar genetic background but differing susceptibilities. A total of 444, 354, 777, and 1768 DE lncRNAs (FDR ≤ 0.05 and |log2FC|≥1) and 170, 153, 169 and 1096 group-specific DE lncRNAs were found in SHR-FI versus SHR-CK, HR-FI versus HR-CK, S-FI versus S-CK and HS-FI versus HS-CK comparisons, respectively. In comparisons of the DE lncRNAs between these four groups, only 48 DE lncRNAs were found to be in common (Fig. 5A). These results suggest that different RILs respond to *Fov* infection in different ways. The number of lncRNAs regulated by *Fov* in the resistant strains HR (354) and SHR (444) was similar (Fig. 5B). However, the number of lncRNAs regulated by *Fov* in S (777) and HS (1768) RILs was much higher than in resistant RILs.

In contrast to the DEGs shown in Fig. 1C, there were more upregulated than downregulated lncRNAs (Fig. 5B,C). The induced expression of a large number of *Fov*-responsive lncRNAs was positively correlated with susceptibility to *Fov* infection (Fig. 5B,C, boxed in blue). By performing Chi-square tests on expressed lncRNAs, we found fewer DE lncRNAs compared with edgeR. The percentage of overlapping DE lncRNAs varied from 13.25% to 39.62% (Supplementary Table S2).

We performed qRT-PCR experiments to verify the expression patterns of lncRNAs in the eight RIL samples. Thirteen upregulated DElncRNAs in response to infection from HS group were chosen for qRT-PCR experiments. The expression patterns of six selected genes are shown in Supplementary Fig. S5. The expression consistency of all 13 genes between their qRT-PCR and RNA-seq results was summarized in Supplementary Table S4. We observed 100% expression consistency for both *Fov*-susceptible RILs (HS and S), supporting the finding that lncRNAs are regulated by *Fov* infection. The expression consistency of these *Fov*-upregulated DElncRNAs was 92.3% and 46.2% consistency for the resistant RILs HR and SHR, respectively, suggesting the genotype-specific response of *Fov* infection.

**Figure 6:**
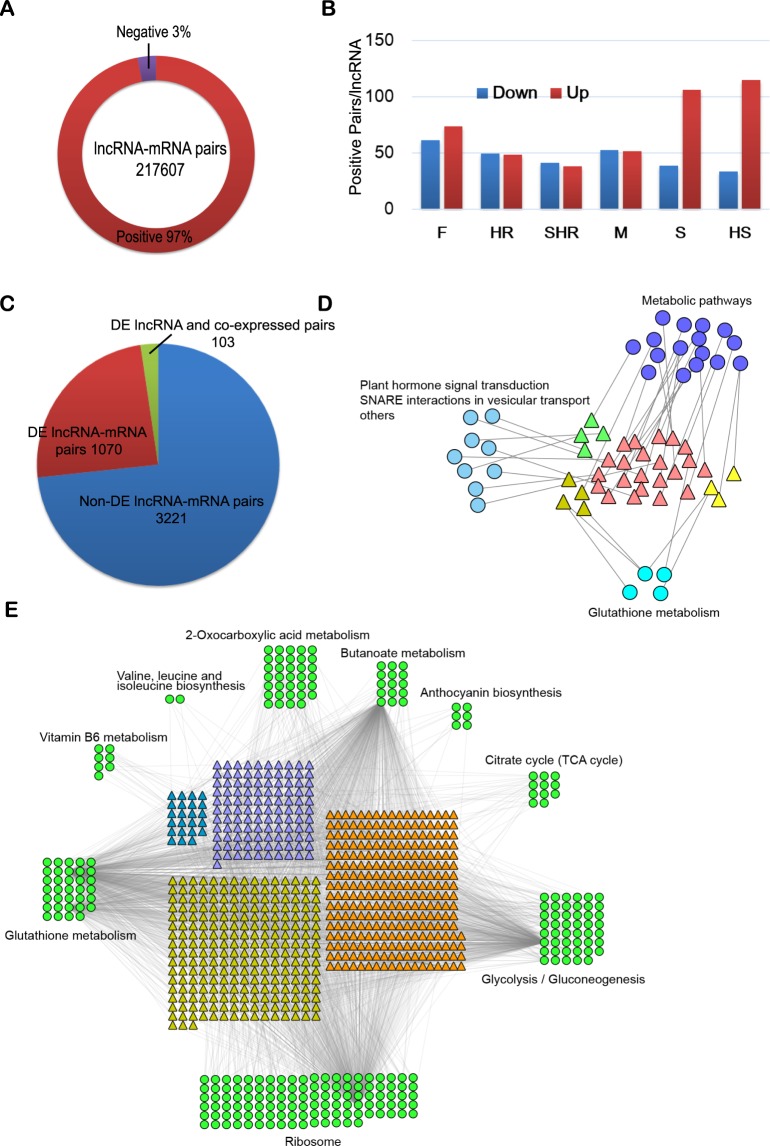
Potential lncRNA target identification and functional analysis. (**A**) Circular plot showing the distribution of lncRNA-mRNA co-expression pairs. The total number of pairs was 217,607. (**B**) Barplot showing the rational distribution of DE lncRNAs involved in negative co-expression pairs. DE lncRNAs were classified as upregulated or downregulated. (**C**) Pie chart showing the relationship between cis-acting and co-expression functional manners. (**D**) Functional network analysis of the potential mRNA targets of DE lncRNAs by cis-acting manner. *Fov*-induced lncRNAs in Fig. 5D are presented. (**E**) Functional network analysis of potential mRNA targets that were co-expressed with DE lncRNAs.

We used k-means clustering to analyze DE lncRNAs and identified eight clusters. The k-means clustering results (Supplementary Fig. S6 and Fig. 5D) corresponded with the results of hierarchical clustering shown in Fig. 6C. Sorting samples by decreasing *Fov* resistance levels, cluster 2, cluster 5, cluster 6, and cluster 8 revealed significantly elevated values after *Fov* infection (Fig. 5D and Supplementary Fig. S6). The reason that these four groups were not clustered together is that their expression values were not at the same level (Y axis in Fig. 5D), although their expression patterns were similar. Cluster 3, cluster 4 and cluster 7 contained 1483 DE lncRNAs and exhibited decreased expression in infected groups, corresponding with the downregulation of DE lncRNAs (Supplementary Fig. S6A).

The distribution of DE lncRNAs on chromosomes was also investigated. In the four comparisons, *Fov*-induced DE lncRNAs were distributed across every chromosome (Fig. 5E). In the SHR-FI versus SHR-CK comparison, more DE lncRNAs were found on chromosomes A02, A03, A07, A08, A11, D11, and D12. In the HR-FI versus HR-CK comparison, more DE lncRNAs were found on chromosomes A03, A05, A11, A12, D02, and D05. In the S-FI versus S-CK comparison, more DE lncRNAs were found on chromosomes A05, A07, A11, D05, D08, and D11. In the HS-FI versus HS-CK comparison, more DE lncRNAs were found on chromosomes A05, A07, A11, D05, and D08. The non-uniform distribution of DE lncRNAs suggests that some genomic locations are enriched in DE lncRNAs. We performed clustering analysis to merge clusters of DE lncRNAs if the distance of adjacent lncRNAs was less than 10 kb. Circular plots revealed obvious clusters on chromosomes A05, A07, A11, D05, and D06 (Fig. 5F), which could serve as genomic hot spots for the response to fungal infection.

### Functional enrichment analysis of pathogen-induced DE lncRNAs

Regulated genes typically show consistent or opposite expression patterns to their regulatory genes^44^ and lncRNAs^45^. We performed co-expression analysis to predict the target mRNAs of DE lncRNAs. After filtering, 211,000 positive and 6607 negative pairs were retained (Fig. 6A). These filtered pairs included 4957 lncRNAs and 21,249 mRNAs, suggesting that potential lncRNA-mRNA regulatory networks are extensive. Of the DE lncRNAs shown in Fig. 5B, most DE lncRNA-mRNA pairs (96.03%) were positive. We then explored the features of each positive pair. The number of positive pairs per lncRNA was much higher for upregulated lncRNAs than for downregulated lncRNAs within the two susceptible RIL groups (paired *t*-test *p* value = 0.03, Fig. 6B), suggesting that some upregulated mRNAs may be regulated by upregulated lncRNAs in susceptible groups.

lncRNAs may function to modulate the transcription of nearby genes in a cis-acting manner^46^. Cis-acting regulation between lncRNAs and mRNAs also occurs. With a threshold distance of 10 kb between mRNAs and lncRNAs, 4394 lncRNA-mRNA cis-acting pairs were identified. Of these, 1173 pairs (26.70%) involving DE lncRNAs were analyzed. Of these 1173 pairs, only 103 (8.78%) passed the co-expression threshold (Fig. 6C), and all were positively correlated, suggesting that there was no significant correlation in expression for cis-acting pairs and that lncRNAs may function in a trans-acting manner more often.

We then analyzed the functional pathways of the co-expressed and cis-acting mRNAs of DE lncRNAs. We focused on upregulated lncRNAs from the four clusters shown in Fig. 5D and Supplementary Fig. S6. First, we investigated the enriched KEGG pathways of mRNAs regulated by lncRNAs in a cis-acting manner. The most highly enriched pathway was the metabolic pathway (Fig. 6D). We also identified other enriched pathways in these four lncRNA clusters, including glutathione metabolism, plant hormone signal transduction, and SNARE interactions in vesicular transport (Fig. 6D). The same analysis was performed for mRNAs co-expressed with lncRNAs. The most highly enriched pathways were ribosome, glutathione metabolism, butanoate metabolism, and glycolysis/gluconeogenesis (Fig. 6E). Interestingly, glutathione metabolism appeared in both cis-acting and co-expression manners, suggesting that it plays an important role in lncRNA regulation in response to *Fov* infection.

## Discussion

*F*. *oxysporum* is an important wilt pathogen that infects over 100 plant species, including cotton, in diverse ecological niches around the world. Breeding and cultivation strategies represent the most promising options to control this pathogen. Very few studies have been conducted to understand the mechanisms underlying *Fov* infection or to collect genetic marker information to facilitate molecular breeding of the important cotton variety sea-island cotton (*G*. *barbadense*). We previously created RILs from two parents selected from the field that showed divergent susceptibility to *Fov* infection. The RILs further diverged in their susceptibility, presenting an attractive opportunity to study sea-island cotton biomarkers of resistance and susceptibility to *Fov*.

In this study, we used a strand-specific RNA-seq approach to profile the transcriptome of parent and RIL plants with divergent susceptibility to *Fov*. We found that *Fov*-upregulated protein coding genes were highly enriched in known disease-resistance pathways, such as oxidation-reduction, glutathione metabolism, anthocyanin biosynthesis, and flavonoid biosynthesis pathways^6,13,47–50^. However, downregulated genes were enriched in different biological functions, which indicates that susceptibility-determining protein coding genes were generally downregulated. Biological terms that were enriched in susceptibility-determining genes included ascorbate and aldarate metabolism. In summary, we obtained a large number of protein coding genes whose expression was responsive to *Fov*-infection and that could be used in molecular breeding in the future. Because of the lack RNA-seq replicates, the statistical power in identification of differentially expressed genes (DEGs) from RNA-seq data between the infected and control cotton for any individual group was limited. Nevertheless, we found that all the *Fov*-regulated genes showed highly similar expression response to *Fov* infection, and a large number of DEGs were overlapped, among multiple cotton genotypes. We therefore suggest the use of the overlapping DEGs as confident *Fov*-regulated genes for future study.

We used qRT-PCR to verify the *Fov*-regulated protein-coding (7) and non-coding genes (13) based on DEGs identified by edgeR analysis in HS group, demonstrating 100% consistency for both mRNA genes and lncRNAs in the same RIL genotype. The validation consistency was close to100% in some other RILs, but not in all. This finding agrees well with the presence of group variations in gene expression and in *Fov* response. Given the genetic difference among these six RILs, it is also possible that the discrepancy might reflect some RIL-specific differences in post-transcriptional regulation of these genes. For example, the presence of the cotton RIL-specific alternative splicing patterns could lead to altered ratio between splice isoforms which would not change the RNA-seq detected mRNA level, but might be sensitive to the specific qRT-PCR primers^51^.

We also performed Chi-square tests to analyze DEGs and DE lncRNAs and compared the results with those of edgeR. The low overlap in genes reflects the essential differences between these methods in calculating DEGs. Because the global expression level of lncRNAs was lower than that of mRNAs, we propose that the sensitivity of Chi-square tests is more dependent on gene expression level, as it detected fewer DE lncRNAs but more DEGs than edgeR analysis.

We also systematically identified sea-island cotton lncRNAs and studied their expression in response to *Fov* infection. In contrast to protein coding genes, lncRNAs showed extensive transcriptional responses to *Fov* infection in the two susceptible RIL plants, whereas resistant RIL plants showed similar transcriptional responses to the two parents. In the susceptible RILs, more lncRNAs were upregulated than downregulated and the number and magnitude of *Fov*-induced upregulated genes were both correlated with fungal susceptibility. Therefore, we identified a set of susceptibility-related lncRNAs. These findings provide additional molecular markers for breeding programs.

LncRNAs play important roles in various biotic and abiotic stress responses in plants by either regulating the transcriptional level of nearby genes in a cis-acting manner or by influencing other genes via trans-acting mechanisms^52,53^. Computational analysis of the two possible types of lncRNA regulation revealed interesting results. Firstly, co-expression analysis of potential trans-acting lncRNA regulatory mechanisms revealed that approximately 97% of lncRNA-mRNA pairs were positively co-expressed, which is much higher than in mammals^45^. It is possible that plant lncRNAs exert less negative and more positive regulation than mammalian lncRNAs. Secondly, protein coding genes co-expressed with DE lncRNAs are highly enriched in plant defense pathways, suggesting the importance of lncRNAs in the plant immune response. Thirdly, cis-acting regulation played a smaller role in modulating mRNA expression patterns, although the mRNAs adjacent to DE lncRNAs were enriched in plant defense-related pathways. Our results suggest that the two methods of lncRNA target prediction are of equal importance to explore lncRNA functions in plant defense. The predicted potential targets and functions of these lncRNAs will need to be verified in future studies.

Historically, a limited understanding of the genome-wide molecular responses of sea-island cotton to *Fov* infection and a lack of molecular markers related to *Fov* susceptibility have hindered the breeding of suitable high resistant cultivars. Transcriptome analysis of RIL plants with divergent *Fov* susceptibilities has revealed differences in mRNAs and lncRNAs that are correlated with fungal susceptibility. Moreover, lncRNA-mRNA network analysis revealed both positive and negative correlations between lncRNAs and their potential trans-acting and cis-acting mRNA partners that are related to disease resistance. Therefore, the findings of this study contribute to our understanding of disease resistance mechanisms in sea-island cotton and provide molecular markers to speed up breeding of disease-resistant cultivars.

## Materials and Methods

### Plant material and pathogen inoculation

One highly aggressive strain of the defoliating fungus *Fusarium oxysporum* f. sp. *vasinfectum* from the College of Agronomy, Xinjiang Agricultural University was used for inoculation.

The seeds of a highly resistant *G*. *barbadense* cultivar (06-146) and a susceptible *G*. *barbadense* cultivar (Xinhan-14) were kindly provided by the Key Laboratory of Agricultural Biotechnology, Xinjiang Agricultural University. Four F_2:6_ RILs were obtained and classified according to their level of resistance to *Fov* infection. These six cotton RILs were grown in germination boxes containing a sterilized mix of peat and sawdust. Before inoculation, we used sterilized scissor to cut fibril of root. Each seedling was inoculated with 10 mL of a suspension of 2 × 10^7^ *Fov* spores per mL by watering injured roots of plants at the two-true-leaf growth stage. The inoculation was performed at room temperature (~25 °C), ≥60% humidity, 16 h/d light, and 8 h/d dark. After 40 h, susceptible cotton seedlings began to wilt. Control plants were not inoculated but were otherwise treated and sampled with distilled water in the same way. Hypocotyls were collected from seedlings for treatment after washing with 75% alcohol and sterile water. All samples were stored at −80 °C for future use.

### RNA extraction and library construction

Total RNA was extracted using a Plant RNA EASYspin Plus Kit (Aidlab, Peking, China) according to the manufacturer’s instructions and treated with RQ1 DNase (Lot M6101, Promega, Madison, United States) to remove DNA. The quality and quantity of the purified RNA were determined by measuring the absorbance at 260 nm and 280 nm using a SmartSpec Plus (Bio-Rad, Hercules, CA, USA). RNA integrity was further verified by electrophoresis using a 1.5% agarose gel. To control for individual variation, we extracted total RNA from three randomly selected biological replicates for each sample and pooled the replicates together for further experiments.

A cDNA library was constructed using 10 μg of total RNA extracted from the hypocotyls of each *G*. *barbadense* sample. Polyadenylated RNA was purified and concentrated with oligo (dT)-conjugated magnetic beads (Invitrogen, Shanghai, China) before being used for directional RNA-seq library preparation. Purified mRNA was iron fragmented at 95 °C followed by end repair and 5′ adaptor ligation. Reverse transcription was then performed with RT primers harboring 3′ adaptor sequences and randomized hexamers. cDNA was purified and amplified, and 200–500 bp PCR products were purified, quantified, and stored at −80 °C until sequencing. cDNA clusters were generated and sequenced using an Illumina HiSeq2000 platform following the manufacturer’s instructions to obtain 50-nt pair-end sequence reads. Two lanes were used for sequencing. Samples were separated by barcodes.

### qRT-PCR

In this study, to evaluate the validity of RNA-seq data, qRT-PCR was performed for the selected DEGs and DE lncRNAs. Polyadenylated RNA was enriched by oligo dT as in RNA-seq, which was then reverse transcribed into cDNA using M-MLV Reverse Transcriptase (Lot R011-01, Vazyme, Nanjing, China) and random primers. qRT-PCR was performed using the StepOne RealTime PCR System (QuantStudio™ 6 Flex Real-Time PCR System Contains the OptiFlex™ Optics System (Applied Biosystems™)) with the SYBR Green PCR Reagents Kit (Lot 11202ES08, Yeasen, Shanghai, China). The PCR conditions consisted of denaturing at 95 °C for 30 s, followed by 40 cycles of denaturing at 95 °C for 10 s and annealing and extension at 60 °C for 30 s. PCR amplifications were performed in triplicate for each sample and normalized using the cotton actin 7 gene as a reference gene. Cotton gene encoding Actin 7 (*ACT7*) was used as an internal control. Data were assessed using the comparative C_T_ (ΔΔC_T_) method^54^, and the relative expression level between the specific gene and *ACT7* was thus obtained and presented. Primers for qRT-PCR analysis are listed in Supplementary Table S5.

### lncRNA identification and classification

By filtering low quality reads and adapters, high quality clean reads were obtained for downstream analyses. These reads were mapped to the Upland cotton genome^34^ using TopHat2^41^ software. Only mapped reads with one genomic location were used for analysis. FPKM was used to evaluate the expression level of genes and lncRNAs. Separated gene models from the same genotype were merged together using the Cuffmerge procedure^55^.

Novel transcripts were detected using Cufflinks and Cuffcompare^56^. Background noise was filtered from novel transcripts based on FPKM (>0.5), length (>200), coverage (>1), and status threshold (OK)^56^. The coding potential capability was calculated using the Coding Potential Calculator^57^ (value < 0). Class code ‘u’ represented lincRNAs, ‘x’ represented long noncoding natural antisense transcripts (lncNATs), and ‘i’ represented intronic transcripts. lincRNA/protein-coding gene pairs were restricted to those that were non-overlapping and 1 kb away from protein-coding genes.

### Identification of common and specific lncRNAs

All separate transcriptome gtf files of *G*. *barbadense* were merged into one gtf file using Cuffmerge with the parameter -g. These merged transcriptomes made it possible to compare the loci of lncRNAs from different sequencing samples using Cuffmerge. The class code ‘u’ represented specific lncRNAs among these sequencing samples. Furthermore, similar sequences were discarded to ensure the reliability of identified specific lncRNAs according to reciprocal BLASTN results with an E threshold value < 1e-10. The class code ‘=’ represented core lncRNAs between *G*. *barbadense* that share fully equal loci. Reciprocal BLASTN analysis (E value < 1e-10) was also run to improve the confidence of identified core lncRNAs, and only those with highly similar sequences were retained for further analysis.

### lncRNA-mRNA co-expression analysis

Based on the expression of each mRNA and lncRNA, correlation coefficients and *p* values were obtained for each lncRNA-mRNA pair. We then filtered the results based on a threshold of absolute correlation coefficient no less than 0.95 and *p* value less than 0.01. In addition to positively correlated pairs, pairs with a correlation coefficient less than 0 were also included. These filtered gene pairs formed the expression network.

### Expression and functional analysis

To measure the pattern of lncRNA expression and screen DE lncRNAs, we applied the edgeR^43^ package, which is specifically designed to analyze differential expression of genes using RNA-seq data. lncRNAs with FPKM < 0.5 in every sample were removed prior to analysis. To identify DE lncRNAs, FC (≥2 or ≤0.5) and FDR (**≤**0.05) cutoffs were applied.

To predict gene functions and calculate the functional category distribution frequency, KEGG analyses were performed using KOBAS bioinformatics resources^58^. Networks were constructed by calculating Pearson’s correlation coefficients for DEGs. Cytoscape (v3.5.1) was used to display the co-expression network^59^. Circos software was used to plot the distribution of lncRNAs in the *G*. *hirsutum* genome^60^.

### Statistical analysis

K-means was used to cluster the differently express pattern genes. Chi-square tests were also used to perform differential expression analysis. All values are presented as mean ± SD. Differences between means were determined using Student’s *t-*tests. *p* values < 0.05 were considered statistically significant. All the statistical analysis was performed by R software.

### Data deposition

Datasets supporting the results of this article are available in the NCBI Gene Expression Omnibus (GEO, http://www.ncbi.nlm.nih.gov/geo/) under Accession Number GSE95288.

## Supplementary information

Supplementary Tables and Figures
