# Supplementary material for: The susceptibility of sea-island cotton recombinant inbred lines to Fusarium oxysporum f. sp. vasinfectum infection is characterized by altered expression of long noncoding RNAs
Source: Sci Rep. 2019 Feb 27;9:2894. doi: 10.1038/s41598-019-39051-2 (PMC6393425; doi:10.1038/s41598-019-39051-2)
Supplement: Supplementary file 1 — Supplementary Tables and Figures [file 41598_2019_39051_MOESM1_ESM.docx]

**The susceptibility of sea-island cotton recombinant inbred lines to *Fusarium oxysporum* f. sp. *vasinfectum* infection is characterized by altered expression of long noncoding RNAs**

Zhengpei Yao^1,4^, Quanjia Chen^1,4^, Dong Chen^2,4^, Leilei Zhan^2,4^, Kai Zeng^3^, Aixing Gu^1^, Jian Zhou^2^, Yu Zhang^2^,Yafu Zhu^1^, Wenwei Gao^1^, Liping Wang^1^, Yi Zhang^2^, Yanying Qu^1^*

^1^ College of Agronomy, Xinjiang Agricultural University, Urumqi, 830052, China

^2^ Center for Genome Analysis, ABLife Inc., Optics Valley International Biomedical Park, East Lake High-Tech Development Zone, 388 Gaoxin 2^nd^ Road, Wuhan, Hubei 430075, China.

^3^ Crop Research Institute, Xinjiang Academy of Agricultural Reclamation, Shihezi, 832000, China

**^4^**These authors contributed equally to this work.

^*^Corresponding Author: Yanying Qu E-mail: xjyyq5322@126.com

**Table S1.** The sequencing and mapping information for all the 12 samples. The clean reads were mapped onto the *G. hirsutum* genome.

| samples | Raw Data | clean data | clean percentage | Total Mapped |
| --- | --- | --- | --- | --- |
| FB_F | 43123599 | 37527023 | 87.02% | 32358524(86.23%) |
| FB_M | 28011347 | 24926855 | 88.99% | 22081458(88.59%) |
| CK_F | 34797116 | 29558952 | 84.95% | 23121193(78.22%) |
| CK_M | 34314178 | 29730916 | 86.64% | 23817179(80.11%) |
| FB_HR | 29663040 | 26391767 | 88.97% | 21923270(83.07%) |
| FB_S | 30728812 | 27133667 | 88.30% | 23495140(86.59%) |
| FB_SHR | 23756929 | 21383877 | 90.01% | 18500622(86.52%) |
| FB_HS | 29800289 | 26321686 | 88.33% | 21910444(83.24%) |
| CK_HS | 34481954 | 30775999 | 89.25% | 25176980(81.81%) |
| CK_SHR | 36721876 | 32662198 | 88.94% | 26085517(79.86%) |
| CK_HR | 42609618 | 38102918 | 89.42% | 30147347(79.12%) |
| CK_S | 33247654 | 28545505 | 85.86% | 22555004(79.01%) |

**Table S2.** The overlapped DE mRNA and lncRNA genes between edgeR and Chi-square test.

| Type | Group | edgeR DEGs | Chi-square DEGs | Overlapped DEGs | Percentage* |
| --- | --- | --- | --- | --- | --- |
| Protein coding Genes | FB_F vs CK_F | 2635 | 2973 | 1054 | 23.14% |
|  | FB_M vs CK_M | 1774 | 3108 | 840 | 20.78% |
|  | FB_HS vs CK_HS | 4850 | 3875 | 1854 | 26.98% |
|  | FB_S vs CK_S | 2775 | 3177 | 1060 | 21.67% |
|  | FB_HR vs CK_HR | 1940 | 2718 | 737 | 18.80% |
|  | FB_SHR vs CK_SHR | 3143 | 3324 | 1208 | 22.97% |
| LncRNA Genes | FB_F vs CK_F | 300 | 136 | 51 | 13.25% |
|  | FB_M vs CK_M | 235 | 132 | 45 | 13.98% |
|  | FB_HS vs CK_HS | 1844 | 852 | 765 | 39.62% |
|  | FB_S vs CK_S | 811 | 266 | 185 | 20.74% |
|  | FB_HR vs CK_HR | 383 | 195 | 98 | 20.42% |
|  | FB_SHR vs CK_SHR | 477 | 181 | 99 | 17.71% |

*Percentage was calculated as the fraction of overlapped DEGs by the total DEGs of these two methods.

**Table S3.** LncRNA filtering steps and the number statistics in each step. The final lncRNAs were classified as intergenic, antisense, and intronic lncRNAs.

| Filtering steps | Isoform Num | Gene Num |
| --- | --- | --- |
| Total transcripts | 82961 | 51657 |
| raw filtered lncRNAs | 68589 | 42148 |
| Distance to gene more than 1000 bp | 63398 | 38741 |
| Removing coding potential transcripts | 41039 | 28106 |
| Removing short transcripts (<200 bp) | 13176 | 11336 |
| Final long intergenic ncRNAs (lincRNAs) | 10868 | 9183 |
| Final antisense lncRNAs | 618 | 519 |
| final_intronic lncRNAs | 1690 | 1634 |

**Table S4.** Summary of the consistency between RNA-seq and qRT-PCR results for the 13 selected lncRNAs

| DE lncRNA | HS | S | HR | SHR |
| --- | --- | --- | --- | --- |
| XLOC_002902 | 🗸 | 🗸 | 🗸 | 🗴 |
| XLOC_025560 | 🗸 | 🗸 | 🗸 | 🗴 |
| XLOC_026370 | 🗸 | 🗸 | 🗸 | 🗸 |
| XLOC_029412 | 🗸 | 🗸 | 🗴 | 🗴 |
| XLOC_029416 | 🗸 | 🗸 | 🗸 | 🗴 |
| XLOC_029437 | 🗸 | 🗸 | 🗸 | 🗸 |
| XLOC_029440 | 🗸 | 🗸 | 🗸 | 🗴 |
| XLOC_031225 | 🗸 | 🗸 | 🗴 | 🗴 |
| XLOC_038804 | 🗸 | 🗸 | 🗸 | 🗸 |
| XLOC_045514 | 🗸 | 🗸 | 🗸 | 🗸 |
| XLOC_045517 | 🗸 | 🗸 | 🗸 | 🗸 |
| XLOC_045582 | 🗸 | 🗸 | 🗸 | 🗴 |
| XLOC_047812 | 🗸 | 🗸 | 🗸 | 🗸 |
| Consistency rate | 100% | 100% | 84.62% | 46.15% |

🗸 represents that the variation tendency of lncRNA expression after *Fov* infection is consistent between RNA-seq and qRT-PCR results. 🗴 represents the tendency was not consistent.

**Table S5. PCR primer table.**

| Gene | Forward | Reverse |
| --- | --- | --- |
| mRNA qRT-PCR Primer | | |
| Gh_D11G2549 | GTGAAACGGCGGAGGAGAT | AGGACTTGTTCAGCAGACCAT |
| Gh_A13G1787 | CCAAGTCCGAAGCCTTCATAC | TCCAGTCATTCAATTCCGTTCA |
| Gh_D10G1825 | GGAAGATGGCGGCTTACCT | TCTTTGATCCACTCCTTGTGTG |
| Gh_D11G1355 | CTTCAGCCGTCTCGTATGTTC | TTAATGCAGAGGTTCGGTACAG |
| Gh_A05G1707 | TCTTCCTCTGCGACAACACT | TTATGGACAATACTGGCGTGAA |
| Gh_A03G1208 | CAGTCGCTCTATCAGGAATCAC | TTGCCAACAGAATGGATCGTAG |
| Gh_D03G1421 | CCACCAGTGTCATCATCATCAA | GAAGGACTTAGTGTTGCTGCTA |
| Gh_D13G2160 | CCAAGTCCAAAGCCTTCATACA | CCAGTCATTGAGTTCCGTTCAT |
|  |  |  |
| *Fusarium oxysporum* gene qPCR Primer | | |
| *FOTG* | AGGTTACGTCCCTGTACTCATG | TCGGATTTGGCAGCATTT |
| LncRNA qRT-PCR Primer | | |
| XLOC_027698 | AAAAGATTCAAAACACAAACAA | GTCTCTCTTTGTTCTTTGATTT |
| XLOC_029412 | GGATCGGTGTAATGATTAACAG | TGCTTTCGCAGTTGTTCG |
| XLOC_029416 | GGCCCCGATGAGTAGGAG | TATTTGCTACAACCACCAAGAT |
| XLOC_029437 | CTACCACATCCAAGGAAGG | CCAATTACCAGACTCAAAGAG |
| XLOC_029440 | CTGTCTACGAGTCGGGTT | CGGTACTTGTTCGCTATCG |
| XLOC_045514 | AACCCTAATTCTCCGTCAC | GCGACGCATCATTCAAAT |
| XLOC_045517 | TATTTAGCCTTGGACGGAAT | ACCAAGAGGCGCTGTCTA |
| XLOC_ 038804 | GCTTTCGCTCAAGACTCGTA | GGCATTAAGGTGGTTGGACTAT |
| XLOC_ 026370 | ATCGCCGAGCTTGGTAAC | CCCATGTTCCATCACCTTAAAG |
| XLOC_ 002902 | TTGATTCAGGCTTGTCCGTTCC | CTCTAAGAACCACGACGCACAA |
| XLOC_ 047812 | TTAATCAAGAACGAAAGCTGGG | GGCATCGTTTATGGTTGAGACT |
| XLOC_ 045582 | GAGGAAGCACTCGCCTGATT | CCAACTGCCAATTCTCAGCC |
| XLOC_ 030864 | TGCCCACGACTATTTCTGTTAA | CAATCCTATTGTGTTGGCCTTC |
| XLOC_ 045536 | TGTGGTTTCGCTGGATAGTAGA | TAATTAGTGACGCGCATGAATG |
| XLOC_ 006445 | CCGGCACCTTATGAGATATCAA | TCAGCCTTGCGACCATACT |
| XLOC_ 025560 | CCTTTCCAGGGAGACAATACC | TGTTGAGGGATATTCGCTCTTT |
| XLOC_ 031225 | GAAGAGTTTGATCCTGGCTCAG | ACGTTCGACTTGCATGTGTTA |


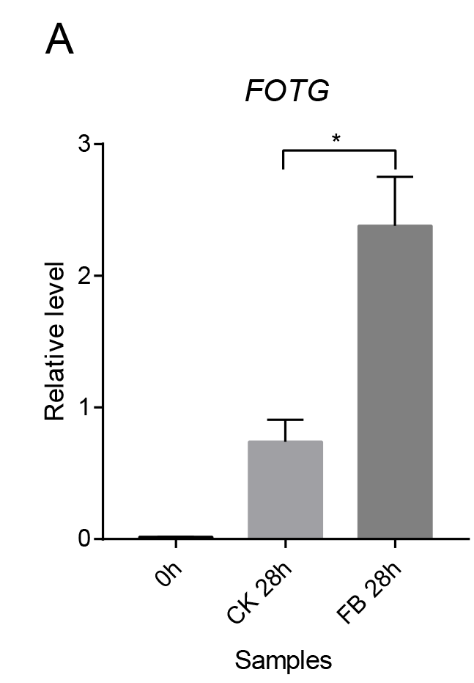


**Supplementary Fig. S1. Evidence of *Fov* infection was obtained by experiments.** (**A**) We performed qPCR to test *Fov* infection. *FOTG* was selected to perform qPCR and showed significant elevated value at 28h of inoculation compared with CK samples.
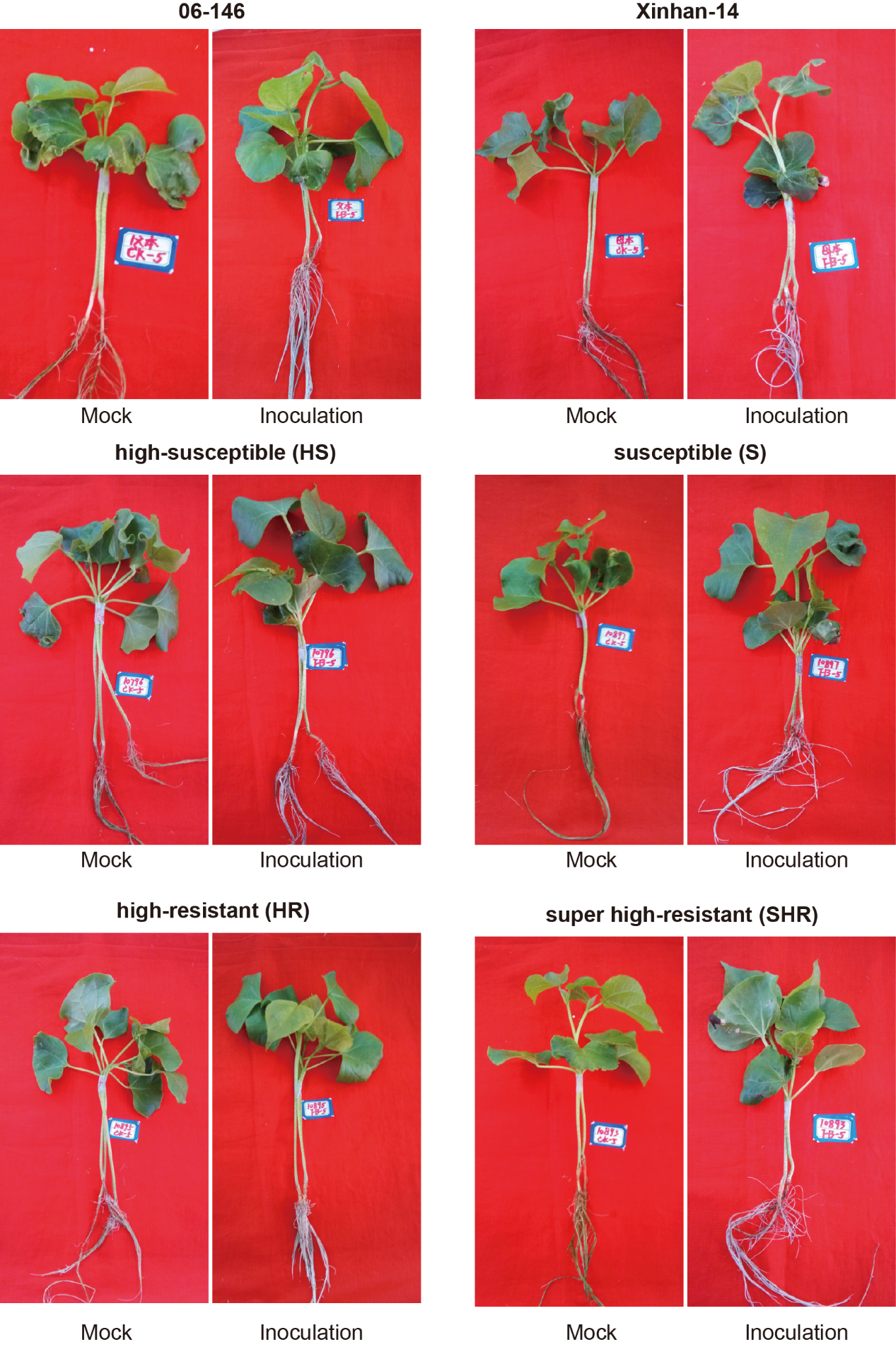


**Supplementary Fig. S2.** Presentation of the characterizations of two *Gossypium barbadense* cultivars (06-146 and Xinhan-14) and their four F_6_ RILs after inoculation with *Fusarium oxysporum* f. sp. *vasinfectum* (*Fov*) for 40h.


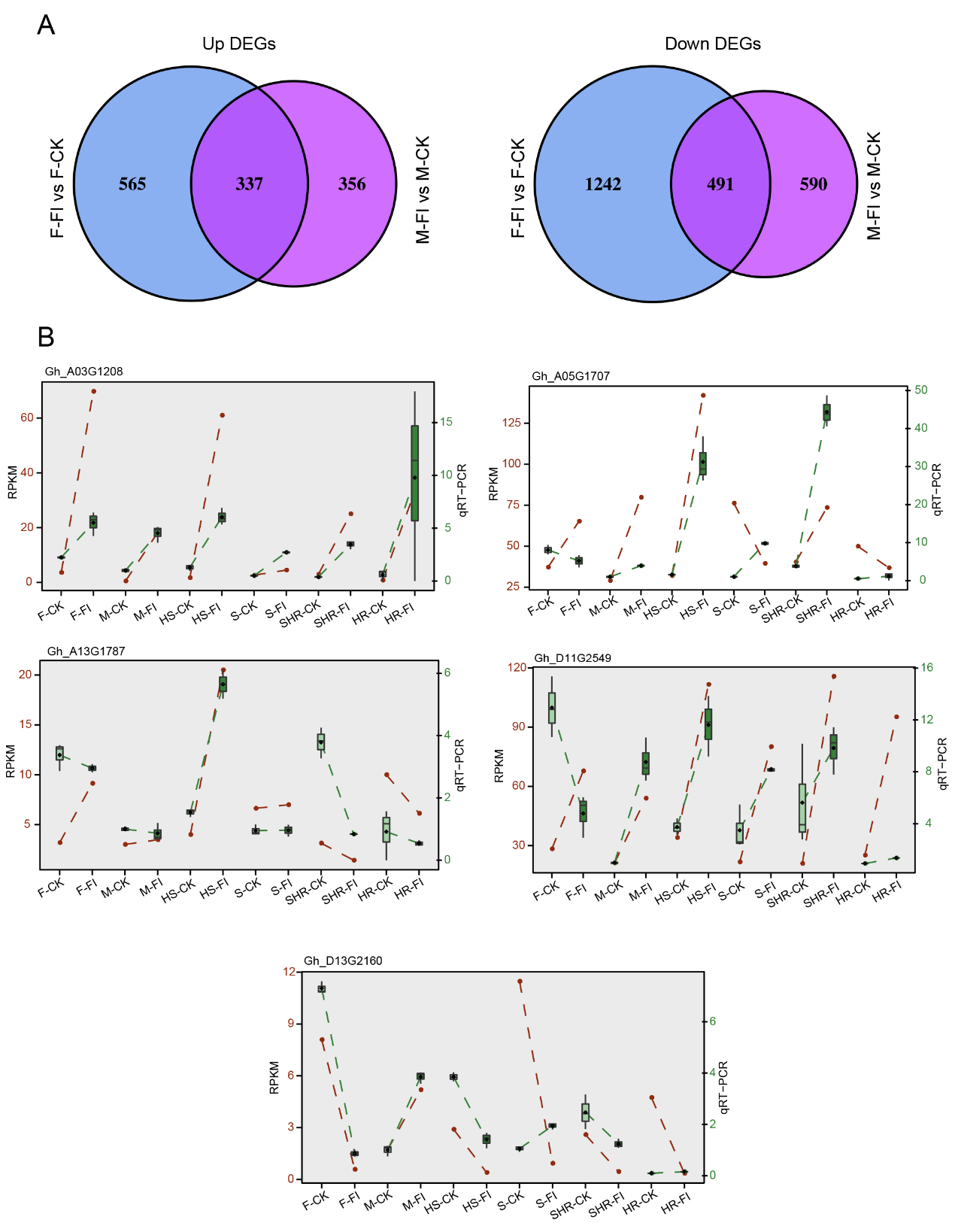


**Supplementary Fig. S3. DEGs analysis and validation for selected DEGs after *Fov* infection.** (**A**) Venn diagram showing the overlapped upregulated (left panel) and downregulated (right panel) DEGs between father and mother groups after *Fov* infection. (**B**) Line plot showing the consistent expression change between RNA-seq (FPKM) and qRT-PCR (Relative level) for five selected DEGs. Three replicates were used to perform qRT-PCR experiment.


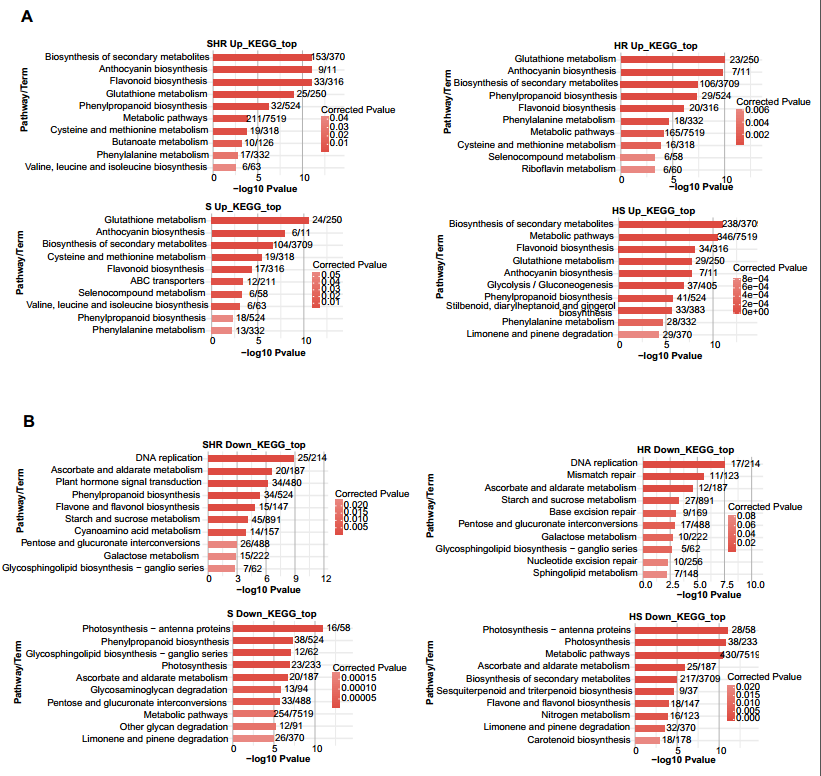


**Supplementary Fig. S4. Functional enrichment analysis for the four DEG groups. KEGG pathways were presented as illustration.** (A) The top 10 enriched KEGG pathways for the up-regulated genes in the four DEG groups. SHR (top left), HR (top right), S (bottom left), bottom right (bottom right) were shown. (B) The same with (A) but for the down-regulated genes in these four groups.


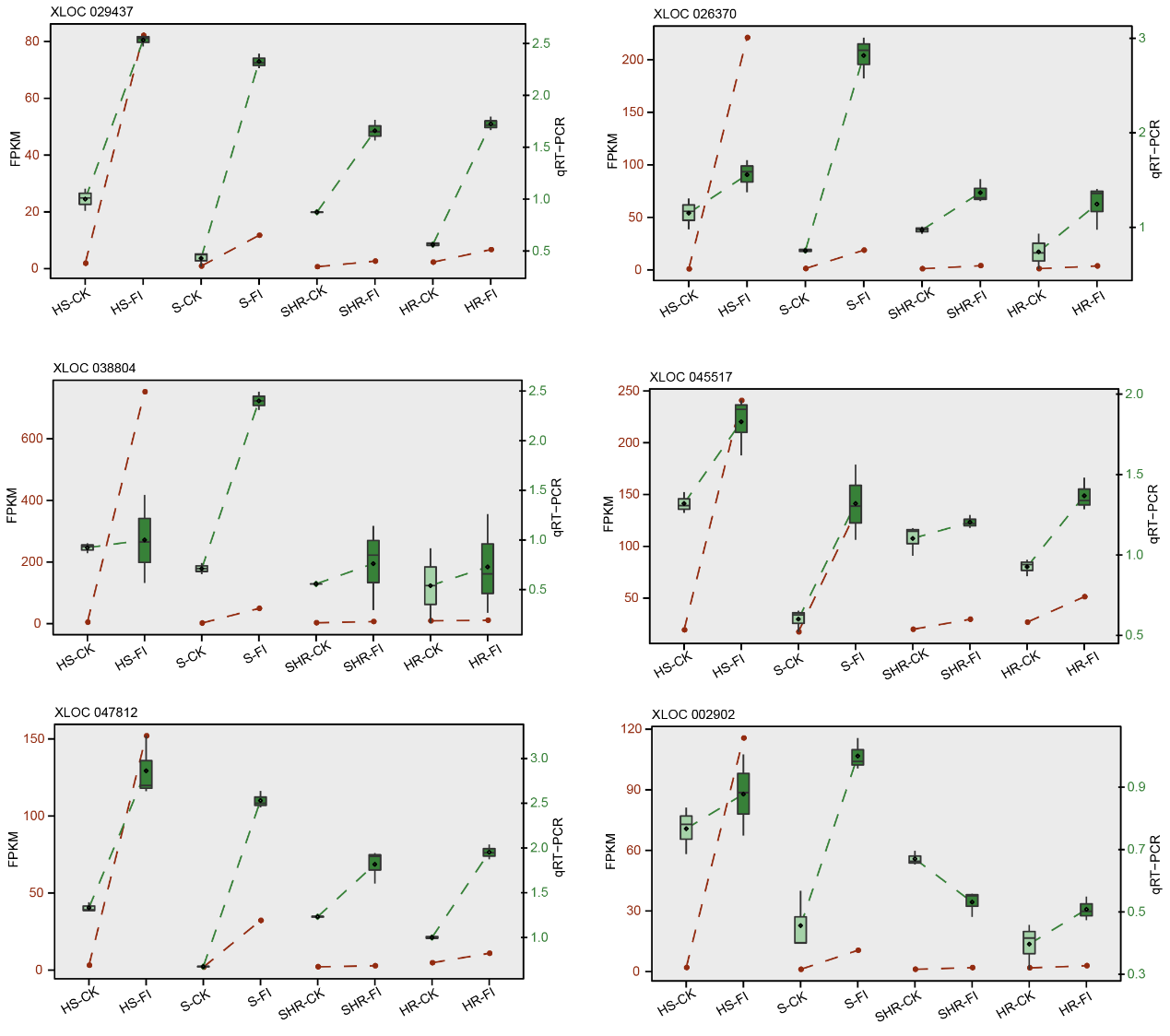


**Supplementary Fig. S5.** Line plot showing the consistent expression change between RNA-seq (FPKM) and qRT-PCR (Relative level) for six selected DE lncRNAs. Three replicates were used to perform qRT-PCR experiment.


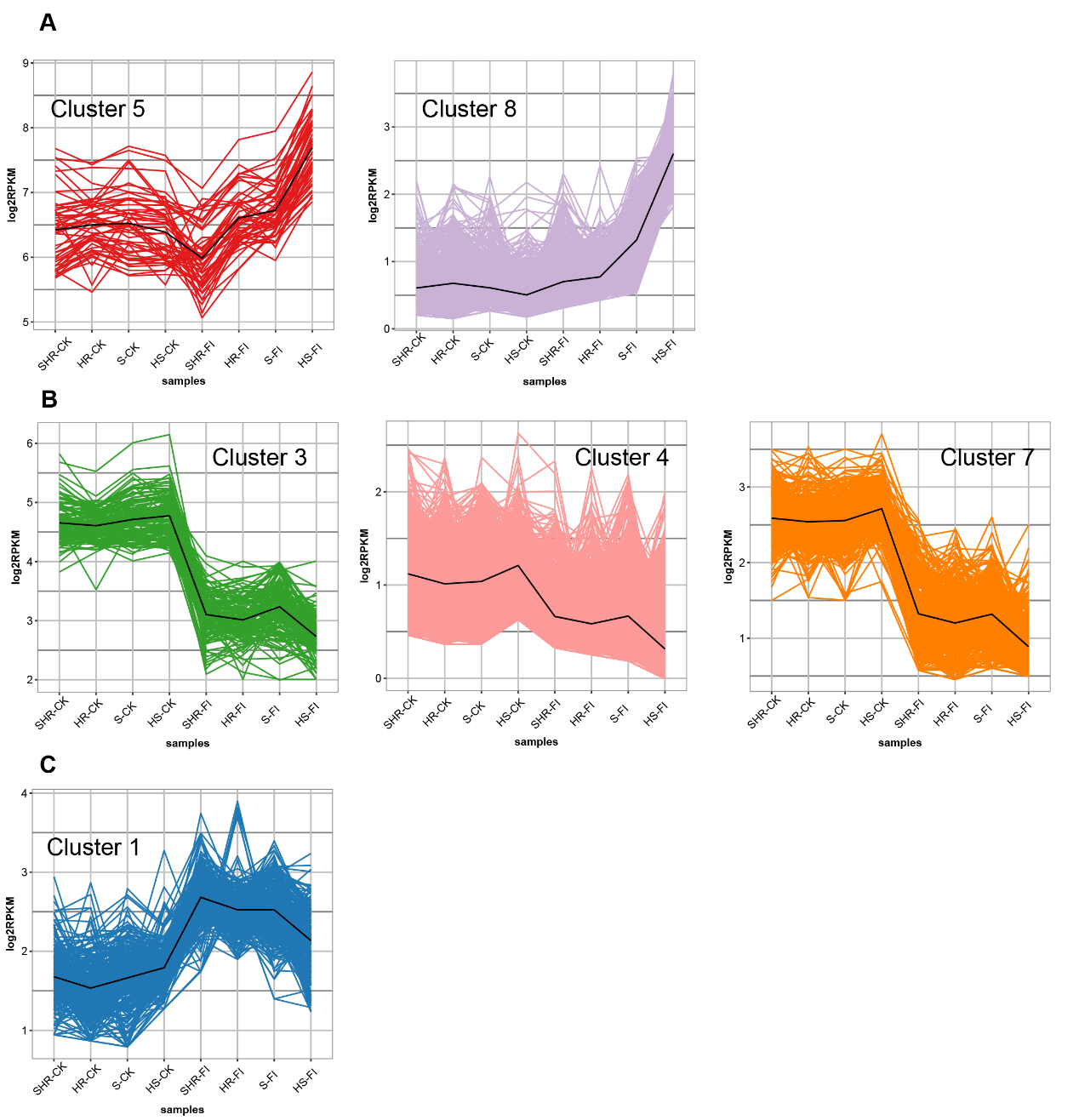


**Figure S6. K-means clustering presentation of the DE lncRNAs.** (A) The expression pattern of DE lncRNAs from cluster5 and cluster8, representing the lncRNAs with same pattern in Figure 6D. (B) The expression pattern of DE lncRNAs from cluster3, cluster4 and cluster7, representing down-regulated lncRNAs after *Fov* infection. (C) The expression pattern of DE lncRNAs from cluster1, representing most up-regulated lncRNAs in SHR and HR groups.
